# The Role of MicroRNAs in Mammalian Fertility: From Gametogenesis to Embryo Implantation

**DOI:** 10.3390/ijms21020585

**Published:** 2020-01-16

**Authors:** Dessie Salilew-Wondim, Samuel Gebremedhn, Michael Hoelker, Ernst Tholen, Tsige Hailay, Dawit Tesfaye

**Affiliations:** 1 Institute of Animal Sciences, Animal Breeding and Husbandry, University of Bonn, Endenicher Allee 15, 53115 Bonn, Germany; dsal@itw.uni-bonn.de (D.S.-W.); mhoe@itw.uni-bonn.de (M.H.); etho@itw.uni-bonn.de (E.T.); thai@itw.uni-bonn.de (T.H.); 2 Animal Reproduction and Biotechnology Laboratory, Department of Biomedical Sciences, Colorado State University, 1351 Rampart Rd, Fort Collins, CO 80523, USA; samuel.etay@colostate.edu; 3 Teaching and Research Station Frankenforst, Faculty of Agriculture, University of Bonn, 53639 Königswinter, Germany

**Keywords:** miRNA, gametogenesis, embryogenesis, mammals, fertility

## Abstract

The genetic codes inscribed during two key developmental processes, namely gametogenesis and embryogenesis, are believed to determine subsequent development and survival of adult life. Once the embryo is formed, its further development mainly depends on its intrinsic characteristics, maternal environment (the endometrial receptivity), and the embryo–maternal interactions established during each phase of development. These developmental processes are under strict genetic regulation that could be manifested temporally and spatially depending on the physiological and developmental status of the cell. MicroRNAs (miRNAs), one of the small non-coding classes of RNAs, approximately 19–22 nucleotides in length, are one of the candidates for post-transcriptional developmental regulators. These tiny non-coding RNAs are expressed in ovarian tissue, granulosa cells, testis, oocytes, follicular fluid, and embryos and are implicated in diverse biological processes such as cell-to-cell communication. Moreover, accumulated evidences have also highlighted that miRNAs can be released into the extracellular environment through different mechanisms facilitating intercellular communication. Therefore, understanding miRNAs mediated regulatory mechanisms during gametogenesis and embryogenesis provides further insights about the molecular mechanisms underlying oocyte/sperm formation, early embryo development, and implantation. Thus, this review highlights the role of miRNAs in mammalian gametogenesis and embryogenesis and summarizes recent findings about miRNA-mediated post-transcriptional regulatory mechanisms occurring during early mammalian development.

## 1. Introduction

Perpetuation and existence of mammalian species are maintained through reproduction. Certainly, at any given time and environmental condition, the reproduction pattern of mammals are believed to be directed by the hereditary codes inscribed during the process of development. More specifically, the genetic material encoded on the gametes are the key determiners, genetic codes/inscriptions written during the formation of one-cell embryo (zygote) determine the fate of embryonic development and the organism’s fitness/destiny concerning the physical and morphological characteristics, reproductive and productive performance, resistance to diseases and adaptability. This general conclusion is deduced based on the assumption of basic dogma of molecular biology, which states that the genetic information is transferred from DNA to RNA to protein. However, after the discovery of non-coding RNAs such as microRNAs (miRNAs) and large non coding regulatory molecules, the central dogma of molecular biology is revisited. For instance, miRNAs, one of the small non-coding RNAs classes, approximately 19–22 nucleotides in length are expressed in a wider ranges of eukaryotes [1]. These tinny non coding RNAs are implicated in the regulation of gene expression either by degrading the messenger RNAs (mRNA) or repression of protein translation [2,3]. With the advancements in DNA and RNA sequencing technology, extensive progresses in genomic and genetic data analysis and annotation methods and current developments in analyzing biochemical pathways and networks, it is becoming clear that along with other species of RNAs, cellular proteins, metabolites, some of the miRNAs are packaged in extracellular vesicles, transported and delivered to neighboring cells [4]. Thus, in addition to cellular miRNAs, extracellular vesicles packed miRNAs are believed to be involved play key roles in gametogenesis and embryogenesis. Therefore, understanding the role and mechanism of cellular miRNAs and extracellular vesicles mediated miRNAs during gametogenesis and embryogenesis provides valuable insights for improving techniques of assisted reproduction and further insights about physiological and communicational events occurring during normal mammalian development or pathological conditions. Therefore, this review highlights the role of cellular and extracellular vesicles originated miRNAs in mammalian gametogenesis and embryogenesis.

## 2. MicroRNAs During Gametogenesis

Oogenesis and spermatogenesis are regulated by complex molecular and physiological events occurring in follicular microenvironment of ovaries and seminiferous tubules of the testis.

Although the final output is generating viable gametes, the processes of oogenesis and spermatogenesis are different in some aspect. For instance, unlike the oogenesis, the processes of spermatogenesis produces four functional spermatids of equal size from a single spermatocyte. Therefore, activation or disappearance of various genes and gene products in the ovarian environment triggers the formation and development of the primordial follicles into anovulatory stage and subsequent ovulation, whereas, expression of genes and the gene products in the testis trigger the production of viable sperms from spermatogonia. Therefore, understanding the role of miRNAs in relation to ovarian and testis physiology is essential to a get a clear overview about posttranscriptional regulatory mechanisms involved in mammalian gametogenesis.

In fact, accumulated evidences have shown that these tinny noncoding RNAs are implicated in various functions including centromere function, silencing of the unpaired sex chromosome during meiosis, and DNA elimination [5]. In developmental processes, miRNAs are involved in cell proliferation and stem cell differentiation [6], developmental timing regulation [7,8], stem cell division [9], spermatogenesis [10], fat and cholesterol metabolism [11] the ovarian environment triggers the formation and development of the primordial follicles into anovulatory stage and subsequent ovulation, whereas, the genes and the gene products in the testis trigger the production of viable sperms from spermatogonia.

### 2.1. The Role of Cellular miRNAs in Follicular Development and Oocyte Maturation

During oogenesis, the interaction between the oocyte and other follicular somatic cells (granulosa and theca cells) leads to the release of matured oocyte with the process of ovulation. Of the follicular somatic cells, granulosa cells involve in oocyte development and maturation by producing oestrogen [12] and trans-linking the bidirectional communication bridge between the oocyte and the theca cells via gap junctions [13,14,15]. Thus, the production of fertilizable ova, which is regulated by the ovarian-uterine-pituitary paracrine and endocrine signaling have been shown distinct gene expression profiles in granulosa, theca and oocytes [16,17,18]. In this regard, transcriptome profile analyses have clearly shown increased expression of genes involved in cell proliferation in the dominant follicle and genes associated with apoptosis and cell death in the subordinate follicles [16,17,18,19].

**Figure 1:**
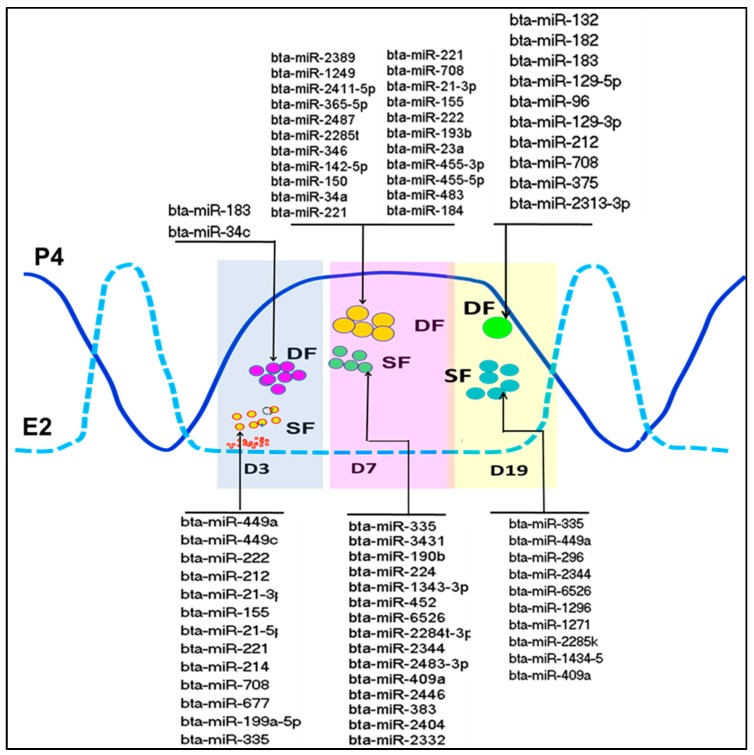
MicroRNAs (miRNAs) enriched in granulosa cells of subordinate (SF) or dominant follicles (DF) on days 3, 7, and 19 of the bovine estrous cycle. Compiled from [31,32]. D = day, P4 = progesterone, E2 = estradiol.

The role of miRNAs in oogenesis has been evidenced by functional knockout of *DICER1* gene [20], argonaute 2 (*AGO2*), a key component of RNA-induced gene silencing complex in miRNA processing machinery, [21] and drosha, a gene responsible for Pri-miRNA processing [22]. These functional studies evidenced the role of miRNA biogenesis for ovarian functionality and progression of gametogenesis. Moreover, several miRNA expression and functional studies have been performed aiming at understanding the basic mechanism of posttranscriptional gene regulation during oogenesis via miRNAs. In this respect, the expression of miRNAs in bovine cumulus–oocyte complexes [23], bovine corpus luteum tissue [24] and adult bovine ovarian cortex [25], cattle fetal ovary [26,27], bovine follicular fluid [28] and embryos [29], bovine granulosa cells [30,31] has been evidenced. Therefore, understanding the fundamental role of miRNAs in relation to the ovarian physiology and in each of the ovarian follicular compartment (oocyte, theca cells, granulosa cells, follicular fluid) during different phases of the female reproductive cycle is required to get an insight into the posttranscriptional regulation of folliculogenesis or oogenesis. Moreover, looking into the miRNA expression patterns in follicles of different size (dominant and subordinate), healthy and atretic could be a step forward towards identification of miRNAs that are potentially involved in follicular development and ovulation. With this respect, previous studies have shown differential expression of miRNAs that are involved follicular cell proliferation, steroidogenesis, luteinization, and oocyte maturation between small and large follicles or between healthy and atretic bovine follicles [31,32,33]. Moreover, miRNAs that are potentially involved in Wnt signalling pathway, Transforming growth factor (TGF-beta) signalling pathway, axon guidance and apoptosis were differential expressed between small and bigger follicles at day 3 of the bovine oestrous cycle (Figure 1) while miRNAs that were involved in metabolism of vitamins, cofactors, lipids, lipoproteins, cysteine and methionine were differentially expressed between small and bigger follicles at day 7 of the estrous cycle [31]. This suggests that miRNAs could be potentially involved in follicular recruitment and selection during the first follicular waves of cattle. Apart from this, understanding miRNA expression patterns during the follicular phase of the estrous cycle would provide a better clue about miRNAs that directly involved in ovulation. With this regard, our previous study have shown miRNAs that potentially involve in cell adhesion, cell proliferation, apoptosis and metabolism were significantly different between the granulosa cells of preovulatory dominant and subordinate follicles at day 19 of the bovine oestrous cycle [32]. Similarly, in zebra fish it is believed that small early vitellogenic follicles are unable to undergo oocyte maturation whereas oocytes in mid- to late vitellogenic follicles can be induced by Luteinizing hormone (LH) to become matured. With this respect, several miRNAs were found to be abundantly expressed in the follicular cells from early vitellogenic and mid- to late vitellogenic stages follicles but the expression patterns of 24 miRNAs including miR-22a-3p, miR-16a, miR-181a-3p, and miR-29a were altered in follicles that were unable to undergo oocyte maturation [34].

Understanding the expression patterns of miRNAs during the different phases of reproductive cycle (luteal or follicular phase) of mammals such as cattle would provide a better insight about post-transcriptional gene regulation underlining follicular atresia and oocyte ovulation [31,32] or to identify miRNAs governing the production of steroid essential for the normal processes of folliculogenesis [35,36,37,38,39]. Nevertheless, aberrant expression of developmentally relevant miRNAs during critical period of the reproductive cycle could negatively affect follicular development resulting for anovulation or ovulation of infertile oocytes. This has been evidence in the study by [40] where the overexpression of miR-378 in the mouse ovary bursa has reduced the ovarian size and the number of pubs.

Several lines of evidences have indicated that during mammalian oogenesis, the oocyte undergoes both cytoplasmic and nuclear maturation accompanied by accumulation of RNAs and proteins. Accordingly, differential expression of miRNAs between mature and immature oocyte have been observed [23,41,42]. For instance, in porcine oocyte, miR-486, miR-10b, miR-10a-5p, miR-183 and miR-21, were increased and miR-210 and miR-27b-3p were decreased as the oocytes developed from GV stage to MII stage [43]. In bovine 30 miRNAs including miR-208a, miR-2317, miR-2320, miR-365-5p, miR-584, miR-628 and miR-876 were exclusively detected in bovine GV oocytes. On the other hand, 35 miRNAs including miR-144, miR-1603, miR-190b, miR-29b, miR-29c, miR-29e, miR-412 and miR-449b were detected in MII bovine oocytes [42]. Similarly, [23] have shown differential expression of several miRNAs including miR-130b between bovine GV and MII stage oocyte. Interestingly, in vitro functional study of miR-130b showed the involvement of this miRNA in oocyte maturation by regulating the *SMAD5* and *MSK1genes* [44]. Similarly, other studies have shown the role of miR-318 [45], miR-202 [46] and let-7, miR-278 [47], miR-378 [48] and miR-125a-3p [49] during oogenesis.

### 2.2. Functional Analysis of miRNAs in Oocyte Companion Somatic Cells

Due to technical difficulties to directly increase or decrease the expression level of miRNAs in oocyte, many of functional analysis of miRNAs with respect to oocyte maturation or folliculogenesis are usually inferred from information gathered after inhibiting or overexpression of certain miRNAs in in vitro cultured granulosa or cumulus cells. In fact, the effects of many of the miRNAs in oogenesis could be manifested at different phases of oocyte development including during either germinal vesicle break down and during the transition of an oocyte in to MII stage through regulating the proliferation and survival of the oocyte companion cells or directly affecting the oocyte itself. For instance, increased miR-378 expression in porcine cumulus cells has impaired cell expansion and the progression of the GV stage oocytes to the MII stage by down regulating the expression of genes associated with cumulus cell expansion [48]. Similarly, increased expression of miR-224 and miR-574 in pig cumulus cells during in vitro oocyte maturation has reduced the proportion of GV oocytes reaching to MII stage [50].

Apart from these, different in vitro functional studies have shown the role of miRNAs in oocyte maturation by affecting the cumulus and granulosa cells. For instance, in vitro functional study has shown that miR-130b promote oocyte maturation by regulating the survival and proliferation granulosa and cumulus cells [44]. Similarly, overexpression or knockdown studies using lentiviral transduction in bovine cumulus cells have shown that miRNAs such as miR-375 could affect oocyte maturation by targeting ADAM metallopeptidase with thrombospondin type 1 motif 1 (*ADAMTS1*) and progesterone receptor (PGR) genes [51]. Moreover, inhibitory role of miR-21-3p in utophagy, which determines the fate of granulosa cells, in bovine granulosa cells by targeting *VEGFA* via PI3K/AKT signaling has been documented [52]. Apart from these, in vitro functional studies have demonstrated that several miRNAs including miR-146a, miR-183-96-182 cluster, miR-383 and others are believed to regulate folliculogenesis or oocyte maturation by maintaining the survival and proliferation of granulosa/cumulus cells functions (Table 1).

### 2.3. The Role of Extracellular Vesicles-Mediated miRNAs in Female Gametogenesis

Oogenesis is a complex processes that involve many aspect of cell-to-cell communications between the oocyte and the oocyte surrounding cells within the follicular microenvironment. This bidirectional communication between the gamete and surrounding somatic cells is mediated by paracrine, autocrine and endocrine signaling factors or by network of gap junctions [71]. Apart from these, the discovery of extra cellular vesicles (EVs) added another dimension on cell-to-cell communications. The study in equine follicular fluid showed that contains microvesicle that carries proteins and miRNAs and some of these miRNAs are present in the surrounding granulosa and cumulus cells suggesting the presence of transfer of bioactive material by microvesicles and exosomes within the follicular microenvironment [72]. The presence of miRNAs in extra cellular vesicles of bovine [28,73], human [74] and porcine [75] follicular fluids indicated the potential roles of EV-mediated miRNAs in folliculogenesis. The expression patterns of the EV-mediated miRNAs depends on follicle size [76] and / or reproductive cycles [77]. For instance, miRNAs that are predicted to be involved in follicle development (miR-99a, miR-100, miR-132, and miR-218) and those which are associated with cellular meiosis (miR-132, miR-212, and miR-214) were detected in EVs of human follicular fluid collected at the time of ovulation [76]. In addition, several evidences have shown predicted and experimentally validated l roles of extracellular vesicles-mediated miRNAs in female gametogenesis. For instance, higher expressions of exosome-derived miR-92a and miR-130b have been detected in follicular fluids of women with oocytes that failed to fertilize [78]. This indicate that miRNAs carried by extracellular-vesicles of the follicular fluid as additional molecules that control stage specific follicular development and oocyte maturation along with the autocrine and paracrine communication occurring in the follicular environment. In addition, some believe that, the release of extracellular vesicle during folliculogenesis is induced by stress condition. For instance, study by [79] demonstrated that the role of exosome-derived from follicular fluid in improving oocyte function by protecting stress during in vitro maturation. Thus, extracellular vesicle which is believed to be released to the surrounding extracellular space during cellular stress condition are believed to contain exosomes, microvesicles, apoptotic vesicles and necrotic debris, which may enhance protective role in the surrounding cells. This has been explained in detail in the work of [80] where co-culture of cumulus oocyte complexes with EVs derived from follicular fluid of cows with heat stress caused relatively higher expression of the *IGFBP2*, *BMP15*, *GDF9*, *HAS2*, and *STAT3* in the cumulus cells compared to those co-cultured in EVs derived from follicular fluids of cows maintained in thermo-neutral condition.

### 2.4. The Role of miRNAs in Male Gametogenesis

Spermatogenesis is a complex process that includes mitotic proliferation of spermatogonia and formation of early spermatocytes, production of haploid round spermatids, chromatin condensation and nuclear shaping, removal of excess cytoplasm and the acrosome and sperm tail formation [81]. The processes of spermatogenesis produce four functional spermatids of equal size from a single spermatocyte and during these processes, certain genes which are expressed to stimulate the production of spermatogenic cells [82]. In fact, the process of male gametogenesis is not only accompanied by phase-specific expression of coding genes but also by expression of array of non-coding genes such as miRNAs [10,81]. With this respect, the presence of miRNAs in canine tests [83], mouse testis [84], murine testis [85] have been detected. Apart from these, increased expression of conserved gonad specific miRNAs, namely miR-202–5p/3p in sertoli cells of testis [86] suggests the possible role of this miRNA in testis development.

Indeed, similar to what has been shown in oocytes, biosynthesis of miRNA during spermatogenesis have been confirmed by developing a *DICER* and Drosha knockout model followed by small non coding RNA sequencing from the Knockout and wild type animals. Interestingly the results from that study have shown 47%–52% of the miRNAs that has been detected in the wild type have been dysregulated in *DICER* and Drosha knockout sperm groups [87] indicating active transcription of miRNAs during spermatogenesis. Moreover, the importance of miRNAs in sperm synthesis and male infertility has been described by analyzing miRNA expression in sperm cells derived from prepubertally hemicastrated Yorkshire boars [88], Landrace and Duroc sperm [89]. On the other hand, altered expression of certain miRNAs could be associated with poor sperm quality and male infertility. For instance, the expression patterns of miR-15a, miR-29b, miR-10a, miR-34a, miR-34b and -34c in sperm cells are associated with male fertility in cattle, human or mouse [30,90,91,92]. Some other studies have also implicated the potential role of different miRNAs in spermatogenesis by regulating the function of the seroli cells, key somatic cells which are very important for testis formation and spermatogenesis [93]. For instance, miR-1285 and miR-762 promote porcine sertoli cells by regulating ring finger protein 4 (*RNF4*) gene expression [94]. Similarly, miR-638 plays roles in pig spermatogenesis by regulating immature Sertoli cell growth and apoptosis by targeting the expression of sperm-associated antigen 1 (*SPAG1*) gene [95].

## 3. The Role of miRNAs during Preimplantation Mammalian Embryo Development

Preimplantation mammalian embryogenesis is the complex process by which the fertilized egg undergoes continues cleavage resulting in the formation of an implantation competent embryo. This early stage embryonic development is marked with the degradation of the maternal stores, minor and major embryonic genome activation, compaction of embryo and formation of embryonic cavity and differentiation of embryonic cells into inner cell mass and the outer epithelial trophectoderm distinct cell linages [96]. Evidences have shown that these key early developmental processes are directed and regulated by the arrays of genes which are expressed in stage specific manner. For instance, when the mouse embryo develops from 2- to 4-cell, from 4- to 8-cell and from compacting embryos to morulae, a total of 717, 831 and 839 genes, respectively showed increasing expression patterns [97]. Similarly, in bovine stage-specific gene expression patterns during preimplantation embryo development [98] and differential expression of about 870 genes including *NANOG*, *SOX2*, and *STAT3*, *ELF5*, *GATA3*, and KRT18 between the inner cell mass and trophectoderm of the bovine blastocyst [99] suggest the presence of dynamic transcriptome profile changes in early stage of mammalian embryos. However, dysregulation of those developmentally relevant genes during any of stages of early embryonic developmental stages could result in abnormal embryo development or lead to early embryonic mortality. In this regard, altered expression of genes that are involved in cell cycle, cell signaling, and energy metabolic pathways was detected in implantation incompetent the dormant mice blastocysts [100]. Moreover, altered expression pattern of genes such as *KRT8*, *PGK1 AKR1B1*, *EEF1A1*, *MSX1, PTTG1*, *PGK1* and *AKR1B1* in bovine blastocysts [101] and *B3GNT5*, *Eomes* and *WNT3A* in human blastocysts [102] could be associated with incompetent embryo development. These and other studies elsewhere indicated that preimplantation embryo development is marked by dynamic and stage specific expression of gene and gene products. However, what triggers the stage specific down regulation or increase expression of certain genes during embryo development remains ambiguous.

Similar to the coding genes, the role of stage specific role of miRNAs during early mammalian embryo genesis is getting attention. The first hint on the role of miRNA in embryo development has been fetched after deletion of miRNA processing genes such as *DICER* [103] and *AGO2* [104] that led to embryonic arrest E6.5 or embryonic death around gastrulation. Thus, it could be speculated that miRNAs may promote or hinder embryonic development by limiting the number and/or amount of proteins or enzymes either by degrading the mRNA or inhibiting protein translation. The role of miRNA in preimplantation embryo development during maternal zygote transition, compaction and blastocysts formation have been evidenced by specific functional studies or miRNA profiling studies. Global degradation of maternally derived miRNAs between the zygote and 2-cell stage followed by de novo synthesis of array of miRNAs 2-cell stage and afterwards, during mouse embryo genesis [105], increased expression of Let-7a until 8-cell stage and a decrease in hatched and intact blastocysts [106], increased expression of miR-130a and miR-21 between zygote and 8-cell stage embryo [107], increased expression of miR-205, miR-150, miR-96, miR-122, miR-146a, miR-145, miR-208 and 496 after bovine minor or major embryonic genome activation [23,29], up regulation of miR-135a, miR-218, miR-335, and miR-449b in the early bovine blastocysts compared to hatched ones [108], down regulation of miR-182 between the 2- and 4-cell stages and its increased level between the 4- and 8-cell stages of mouse embryo [109] are some examples that indicate the dynamic nature of miRNAs expression during preimplantation embryo genesis. Moreover, results obtained from mouse and zebra fish have shown the presence of certain miRNAs including the let-7 family that are maternally inherited and dispensable for early embryonic development [105,110]. This suggests an exquisite stage specific expression of miRNA during early embryo development. Nevertheless, it should be also noted that although several miRNAs may dispense their function in stage specific manner to modulate developmental transitions, some miRNAs may exhibit no or little changes between the developmental stages and these miRNAs could have a house keeping role during key developmental processes.

The expression patterns of miRNAs during preimplantation period could also indicate embryonic development abnormalities. For instance, increased level of miR-145 has been shown in embryos produced by somatic cell nuclear transfer, which usually are weak in development. Moreover increased expression of miR-24 was associated with lower blastocyst yield [111]. Thus, suppression of the expression of miRNAs with deleterious impact could increase embryonic development and embryo malformation. With this respect reducing miR-145 expression was found to increase the expression of pluripotency genes such as *POU5F1* and *SOX2*, and subsequently improved the blastocyst yield [112]. On the other hand, the expression of certain miRNAs in the embryo proper is beneficial for further embryo development. For instance, reducing the expression level of miR-130b in bovine preimplantation embryo development [44] and downregulation of miR-302, which possibly regulate maternal transcript clearance during embryogenesis could indicate low embryonic developmental competence [113].

### 3.1. The Role of Embryonic miRNAs in Embryo Implantation

Once the embryo undergoes cell linage specification and differentiation, it attaches and implants in the uterus. This process is believed to be epigenetically regulated both at the transcriptional and posttranscriptional levels. It is well established that the processes of embryonic development is progressed by temporally and spatially established bidirectional cross-talk between the embryo and the maternal environment. During the first few phases of developmental processes, the embryo is challenged by the immune system, the biochemical composition and concentration of the oviduct and endometrial environment. Therefore, normal embryonic development occurs when the immune system of the mother triggered during the time of pregnancy is suitable for the mother and developing embryo [114]. Thus, embryonic development and implantation is manifested by the synergetic effect of both the embryonic and maternally originated endocrine, paracrine and autocrine modulators and molecules such as cadherins, selectins, and integrins, cytokine, growth factors and their receptors and chemokines, see review [115].

It is well known that as the embryo approaches to the stage of implantation the embryonic blastomeres undergo an apical-basal polarization which eventually segregate into two distinct cell populations called the trophectoderm, the outside cell population, and the inner cell mass, the inner cell population are formed at the blastocysts stage. Although the information is scare, miRNA are thought to be involved in these key developmental processes, namely embryonic cell segregation and development of embryonic outgrowth and subsequently implantation. Dysregulation of miRNAs during these critical steps of embryo development could affect cell differentiation and subsequently embryo implantation. In this respect, *DICER* knockout experiments showed that miRNA may maintain pluripotency of epiblasts by inhibiting apoptosis and maintain trphpblast stem cell by blocking the activity of *Cdkn1a* (p21) and *Cdkn1c* (p57) genes [116]. Moreover, previous study has also indicated detection of miRNA in first trimester and term trophoblast cells, but the expression of certain miRNAs was found to be different between these cells suggesting that the involvement of miRNAs in different trophoblast cell characteristics [117]. Similarly, elevated expression of miR-106a in the inner cell mass and a restriction expression of miR-93 on the trophectoderm and the future primitive endoderm suggests the possible role of these miRNAs in embryonic differentiation [118]. Moreover, differential expression of 526 miRNAs including let-7b, miR-23a, miR-27a, miR-291a, miR-425, miR-429 was observed between the embryonic outgrowth and the blastocyst stage embryos [119]. Similarly, differential expression of certain miRNAs between 8-cell stage embryos and trophoblast tissue [120] is additional indication of the fact that miRNAs of embryonic origin could adjust their expression for selective regulation of genes that are required for implantation. Nevertheless, the expression of certain miRNAs in the embryo could hinder embryo implantation. With this respect, previous study has demonstrated altered expression of several miRNAs including let-7a, -7d, -7e, -7f and -7g in the dormant blastocysts compared to the activated blastocysts counterpart [106,121].

### 3.2. The Role of Maternal miRNAs in Embryo Implantation

Along with implantation competent embryo, maternal receptivity is required for embryo implantation and successful pregnancy establishment. Temporally and spatially expressed genes in the uterine epithelial and stromal cells in response to changes to progesterone and prostaglandins hormones and release of embryonic signals such as interferon by the embryo may influence uterine receptivity and embryo implantation in mammals [122] For instance, the expression patterns of cadherin, selectins, and integrin, cytokine, growth factors and their receptors, cytokines and their receptors as key molecules that could be required for pregnancy establishment in mammals [123,124,125,126]. During embryo implantation, cell adhesion and motility is regulated by the interaction of integrins expressed on the trophoblast cell surface and the extracellular matrix proteins located on the endometrium [127,128,129]. However, during this period, the endometrium is not only secreting integrins and adhesion molecules, but also several endometrial molecules such as miRNAs. Thus, understanding miRNA-mediated posttranscriptional regulatory mechanism of embryo implantation will pave the way towards identifying non-coding molecular markers of endometrial origin, which could be potential indicators of pregnancy establishment. For instance, miRNA expression profile in endometrial epithelium of normal fertile woman during menstrual cycle differential expression of miRNAs in the window of implantation (days 19–23), early proliferative, late proliferative, early secretory, and late secretory (24–28) phases has been detected in endometrial epithelium of normal fertile woman during menstrual cycle [130]. In addition, differential expression of endometrial miRNAs associated with cell adhesion and cell cycle related pathways during the secretory phase of the endometrium in women with repeated implantation failure [131] and differential expression of miRNAs associated with cell cycle, cell death, cell adhesion, and metabolism between receptive and perceptive phase of human endometrium [132]. These results could suggest the endometrium microenvironment of mammals could enhance or switch off certain miRNAs in response to its n morphological and functional transformation from the prereceptive to the receptive phases.

Embryo implantation is indeed the key process in pregnancy establishment and the embryo and the maternal environment each contribute specific molecules that make the uterus hospitable to the embryo. Indeed, the “cross-talk” between the embryo and the uterus involves the expression of a diverse array of genes. For instance, differential expression of 518 and 374 genes between implantation sites and inter-implantation sites in mouse and rat uterus, respectively suggests the induction or repression of arrays of endometrial genes at the site of embryo attachment [133].

As a single miRNAs targets several genes and the information gathered from one miRNA could represent the function of several genes, identification of key miRNA signatures involved in embryo-maternal cross-talk is one of the molecular analysis approaches that could be employed to uncover the molecular mechanisms underlying pregnancy establishment in mammals. With this respect, previous study using Exiqon miRCURY LNA Arrays identified increased expression of 13 miRNAs including let-7a, let-7c, let-7d and let-7b and decreased expression of miR-290-5p and miR-292-5p in the implantation site compared to the inter-implantation site in the pregnant mouse uterus at day 5 of the gestation period [134]. Similarly, another study has identified about 72 differential expression miRNAs including miR-96, miR-30b, miR-290-3p and miR-762 between the implantation site compared to inter-implantation sites at day 5 of the gestation period in mice using Exiqon miRCURY LNA Arrays [135].

Similarly in cattle several miRNAs including miR-3902-3p, miR-1825, miR-885-3p, miR-504-3p, miR-92b and miR-31b were reported between high and low receptive animals [136]. In pigs, endometrial miRNAs, which are associated with angiogenesis, proliferation, and tissue remodeling were differentially expressed between highly prolific Chinease Erhualia, and Landrace×Large Yorkshire at day 12 of the gestation period [137]. These results are indicating that miRNAs may be involved in the maintenance and establishment of embryonic survival and embryonic implantation during mammalian embryo development. Moreover, individual functional assay revealed endometrial miR-143, which was upregulated during implantation phase in rat endometrium due to blastocysts activation and uterine decidualization, is believed to be involved embryo implantation by promoting cell proliferation and invasion [138]. Interestingly, miRNA of endometrial origin not only regulate embryo implantation by regulation of endometrial cells, but also could be taken by the embryo to activate embryonic genes. With this respect, in vivo and in vitro studies by [130] have demonstrated the uptake of endometrial miR-30d by embryo trophoectoderm, which in turn triggered the expression of genes associated with embryo adhesion. Interestingly, another study has also shown poorer implantation rates when miR-30d knockout embryos were transferred to miR-30d knockout recipients [139]. Apart from these, several gain and loss of functional studies in endometrial miRNAs have evidenced the potential role different miRNAs such as miR-200a [140], miR-429 [141] and miR-145 [142] in embryo implantation and development [142], (Table 2).

### 3.3. The Role of Extracellular Vesicle Mediated miRNAs in Embryo—Maternal Communication

Although several cellular originated molecules are believed to be essential for successful pregnancy establishment, understanding the mechanism by which the two parties communicate each other using molecular messengers will pave the way towards identifying molecular markers associated with pregnancy establishment. Since the extracellular vesicle (EVs) can cross the physiological barriers, these molecular cargos are believed to be involved in cell-to-cell communication and also able to modulate the immune response under normal and pathological conditions [153]. Thus, the EVs of embryonic, oviduct, or uterine origin interact to promote embryonic growth, differentiation and implantation [154]. For instance, the oviduct is the key female reproductive organ where gamete transport, fertilization and early embryo development occurs and the success of all these developmental events in the oviduct depend on a bidirectional dialog that could possibly established between the oviduct the gametes/embryo [155]. In the bovine oviductal fluid [156] has identified EVs at postovulatory-stage, early luteal phase, late luteal phase and pre-ovulatory stage and those EVs carried miRNAs, different types of non-coding RNAs and several proteins clusters, which may be implicated in preimplantation embryo development and gamete-oviduct interactions. Apart from these, oviductal EVs are implicated in maintaining sperm Ca2+ homeostasis, sperm viability, sperm capacitation and the acrosome reaction [157] and packaging and delivering elevated levels of PMCA1 to the sperm [158]. In vitro study has also shown the role of oviductal extracellular vesicles in improving embryonic quality and modifying the expression of developmentally important genes [159]. Thus, based on the findings outlined in this review and results accumulated somewhere, EVs of oviductal origin could play vital roles in regulating female fertility by enhancing fertilization rates and by promoting early preimplantation embryo development.

Apart from their role during fertilization and early development occurring in the oviductal environment, potential key roles of EVs during in utero embryo development and implantation have been stated in several occasions. Indeed, once the embryo enters the uterus, uterine, embryonic or trophoblast cells origin EVs could mediate the adaptation of the embryo to the maternal environment. The study of [160] revealed the presence of miRNA containing EVs in the implantation site suggesting their role during implantation processes of mammalian embryo development. However, during the later stages of pregnancy just after the formation of placenta, EVs of the placenta origin would be the main players to maintain the already established pregnancy, see the review by [153]. For instance, in human it has been found that at later stage of pregnancy placenta secretes exosomes carrying bioactive Fas ligand (*FasL*)- and a TNF superfamily member gene named as *TRAIL* to tie up the immunomodulatory and protective role of placenta indicating EV-mediated immune modulation and survival mechanism for the fetus in the uterus [161]. This may indicate that extracellular mediated molecules at the embryo-maternal or feto-maternal junction are additional molecular messengers that regulate stage specific embryonic development that modulate embryo-maternal cross talk.

## 4. Conclusions

Understanding the molecular mechanisms underlining gametogenesis and embryo–maternal interaction requires identification of molecular signatures associated with these processes to pave the way towards the identification of markers of successful pregnancy. Thus, in addition to miRNAs, several molecular approaches such as structural genomics, functional genomic approaches, and integrated analysis of transcriptome and epigenome (DNA methylation and histone modification) using high-throughput next generation sequencing techniques are essential to better understand and identify genomic regions associated with pregnancy establishment and fertility.

## Figures and Tables

**Table 1:** List of miRNAs That Are Associated with Granulosa, Cumulus, And Oocyte Development.

MiRNA	Function	Author/s
miR-202	Oogenesis and fecundity in fish	[46]
let-7 miR-278	Ovarian arrest and blocked oocyte maturation in adult insects	[47]
miR-318	Oogenesis in drosophila	[45]
miR-224	Porcine oocyte maturation	[50]
miR-378	Porcine oocyte maturation and mouse in vivo follicular development and oocyte in vitro maturation	[40,48]
miR-274	Oocyte maturation in pig	[53]
miR-375	Bovine oocyte maturation	[51]
miR-125a-3p	Mouse oocyte germinal vesicle breakdown	
miR-21-3p	Inhibits bovine granulosa cell autophagy	[52]
miR-383	Cell proliferation in bovine granulosa in mice	[54]
miR-183-96-182 cluster	Promote bovine granulosa cells proliferation and cell cycle transition	[55]
mir-130b	Bovine granulosa and cumulus cell proliferation and oocyte maturation	[44]
mir-17-92 cluster	Bovine granulosa cell proliferation and differentiation	[56]
miR-424/503	Bovine granulosa cell proliferation and cell cycle progression	[57]
miR-375	Proliferation and apoptosis in bovine cumulus cells	[58]
miR-31 miR-143	Regulate steroid hormone synthesis and inhibit bovine granulosa cells apoptosis by targeting follicle stimulating hormone receptor (*FSHR*) gene	[59]
miR-125b	Regulates granulosa cells apoptosis in the yak ovary	[60]
miR-21	Prevented apoptosis via the *PI3K/Akt* signaling in bovine cumulus cells	[61]
miR-96	Regulates and progesterone production and luteal development in human luteinized granulosa cells	[62]
miR-335-5p	Regulates human granulosa cells proliferation by targeting serum/glucocorticoid regulated kinase family member 3 (*SGK3*) expression	[63]
MiR-99a	Regulation of granulosa cell proliferation and apoptosis in woman affected by polycystic ovary syndrome PCOS	[64]
miR-143	Promotes follicle-stimulating hormone (FSH) -induced estradiol production and granulosa cell proliferation in humans	[36]
miR-181a	Regulate oxidative stress-induced Forkhead box protein O1 (*FOXO1*) acetylation and granulosa cell apoptosis in mice	[65]
miR-23a. miR-27a	Apoptosis in human granulosa cells	[66]
miR-15a	Regulates cell proliferation and steroid hormone production in human ovarian granulosa cells	[67]
miR-126-3p	Promoting cell proliferation and inhibiting cell apoptosis of porcine granulosa cells	[68]
miR-92a	Regulates porcine ovarian granulosa cell apoptosis	[69]
miR-1275	Implicated in porcine granulosa cell apoptosis and estradiol production	[70]
miR-378	Estradiol production in porcine granulosa cells	[35]

**Table 2:** List of miRNAs That Are Associated with Embryo Development and Embryo Implantation.

miRNAs	Potential Function	Author/s
miR-143	Human endometrial stromal cells cell proliferation, migration and invasion	[138]
miR-193	Regulation of implantation rate in mouse uterus	[143]
miR-181	Inhibit mouse embryo implantation	[144]
miR-141	Mouse endometrial cell proliferation and apoptosis	[145]
miR-451	Regulate embryo implantations sites	[146]
miR-429	Mouse embryo implantation by suppressing epithelial-mesenchymal transition	[141]
miR-29b	Bovine somatic cell nuclear transfer (SCNT) embryo development	[147]
Let-7a	Implantation of the activated mouse blastocysts, regulation of mouse blastocyst attachment and outgrowth	[106,121]
miR-34a	Suppress human trophoblast cell invasion	[148]
MiR-520	Regulating human trophoblast cell apoptosis	[149]
miR-126a-3p	Determining the number of implantation sites in murine endometrium	[150]
miR-22	Regulates embryo implantation in mice by targeting Tiam1/Rac1 pathways	[151]
miR-199	Promote rat endometrial stromal cells cell proliferation and apoptosis	[152]
miR-200a	Regulation of implantation rate in mouse uterus	[140]
